# Supplementary material for: Highly Sensitive and Selective Formaldehyde Gas Sensors Based on Polyvinylpyrrolidone/Nitrogen-Doped Double-Walled Carbon Nanotubes
Source: Sensors (Basel). 2022 Nov 30;22(23):9329. doi: 10.3390/s22239329 (PMC9739274; doi:10.3390/s22239329)
Supplement: Supplementary file 1 [file sensors-22-09329-s001.zip › sensors-2012300-supplementary.pdf]

Table S1. Based on experiment study of pristine CNTs and their functionalization or composite materials for formaldehyde detection.

Samples	Working Temperature	Conc. (ppm)	Sensor response	LOD	Note	Ref.
SnO_2<br><br>MWCNTs-doped SnO_2<br><br>MWCNTs-doped SnO_2	125-375 °C<br><br>125-375 °C<br><br>250 °C	50<br><br>50<br><br>50<br>0.05	(R_{gas}/R_{air})<br><br>1.0-1.1<br><br>1.25-1.50<br><br>~3.75<br>~1.0	-<br>-<br><br>0.05 ppm	MWCNTs-doped SnO_2 sensor enabled 3-fold improved compared with undoped SnO2 sensor at 250 °C. The drawback of metal oxide-based gas sensors requires operation at high temperature for high performance working	1
ZnO/MWCNTs	RT	10	($\Delta R/R_0$)×100 %<br><br>~0.02	10	Although the sensor can operate at room temperature, but the response is very low	2
Pure MWCNTs<br><br>Amino group-MWCNTs	RT<br><br>RT	0.02-0.2<br><br>0.02-0.2	($\Delta R/R_0$)×100 %<br><br>~0.1-0.2<br><br>~1.7-5.4	0.02	Amino group-MWCNTs sensor enabled 3-13-fold improved compared with pure MWCNTs sensor at room temperature. The preparation process of functionalization of MWCNTs with amino groups involved with the numerous functionalization methods (e.g., refluxing, evaporating and centrifugation for several times), which is not economically attractive.	3
DWCNTs<br>PVP/DWCNT<br>N-DWCNTs<br>PVP/N-DWCNTs	RT	1000-5000<br>1000-5000<br>250-5000<br>250-5000	($\Delta R/R_0$)×100 %<br><br>0.20-2.28<br>0.23-4.53<br>7.83-19.82<br>9.50-33.72	506<br>410<br>115<br>15	- Can operate at room temperature<br>- Do not involve various functionalization method for N-DWCNTs	This work

Room temperature (RT)

References

[1] Wang, J.; Liu, L.; Cong, S.Y.; Qi, J.Q.; Xu, B.K. An enrichment method to detect low concentration formaldehyde. *Sens. Actuators B* **2008**, 134, 1010-1015.

[2] Xiang, J.; Singhal, A.; Divan, R.; Stan, L.; Liu, Y.; Paprotny, I. Selective volatile organic compound gas sensor based on carbon nanotubes functionalized with ZnO nanoparticles. J. Vac. Sci. Technol. **2021**, 39, 042803

[3] Xie, H.; Sheng, C.; Chen, X.; Wang, X.; Li, Z.; Zhou, J. Multi-wall carbon nanotube gas sensors modified with amino-group to detect low concentration of formaldehyde. *Sens. Actuators B* **2012**, 168, 34-38.
